# miR375-3p Distinguishes Low-Grade Neuroendocrine From Non-neuroendocrine Lung Tumors in FFPE Samples

**DOI:** 10.3389/fmolb.2020.00086

**Published:** 2020-05-19

**Authors:** Simone Detassis, Valerio del Vescovo, Margherita Grasso, Stefania Masella, Chiara Cantaloni, Luca Cima, Alberto Cavazza, Paolo Graziano, Giulio Rossi, Mattia Barbareschi, Leonardo Ricci, Michela Alessandra Denti

**Affiliations:** ^1^Department of Cellular, Computational and Integrative Biology (CIBIO), University of Trento, Trento, Italy; ^2^Division of Anatomical Pathology, Santa Chiara Hospital, Trento, Italy; ^3^Unit of Surgical Pathology, AUSL/IRCCS, Reggio Emilia, Italy; ^4^Unit of Pathologic Anatomy, Forlanini Hospital, Rome, Italy; ^5^Operative Unit of Pathological Anatomy, Azienda USL della Romagna, Hospital Santa Maria delle Croci, Ravenna, Italy; ^6^Department of Physics, University of Trento, Trento, Italy; ^7^CIMeC, Center for Mind/Brain Sciences, University of Trento, Rovereto, Italy

**Keywords:** neuroendocrine, microRNA, biomarker, lung cancer, miR-375

## Abstract

Lung cancer is still one of the leading cause of death worldwide. The clinical variability of lung cancer is high and drives treatment decision. In this context, correct discrimination of pulmonary neuroendocrine tumors is still of critical relevance. The spectrum of neuroendocrine tumors is various, and each type has molecular and phenotypical differences. In order to advance in the discrimination of neuroendocrine from non-neuroendocrine lung tumors, we tested a series of 95 surgically resected and formalin-fixed paraffin embedded lung cancer tissues, and we analyzed the expression of miR205-5p and miR375-3p via TaqMan RT-qPCR. Via a robust mathematical approach, we excluded technical outliers increasing the data reproducibility. We found that miR375-3p levels are higher in low-grade neuroendocrine lung tumor samples compared to non-neuroendocrine lung tumors. However, miR375-3p is not able to distinguish among different types of neuroendocrine lung tumors. In this work, we provide a new molecular marker for distinguishing non-neuroendocrine from low-grade neuroendocrine lung tumors samples establishing an easy miRNA score to be used in clinical settings, enabling the pathologist to classify more accurately lung tumors biopsies, which may be ambiguously cataloged in routine examination.

## Introduction

Pulmonary neuroendocrine (NE) tumors form a distinct group of neoplasms that share morphological, immunohistochemical, ultrastructural, and molecular features. The clinical spectrum is various, from low-grade typical carcinoid (TC) and intermediate-grade atypical carcinoid (AT) to high-grade large cell NE carcinoma (LCNEC) and small cell lung carcinomas (SCLC). Currently, the 2015 World Health Organization classification of pulmonary NE tumors is based on combined architectural patterns with the two most relevant parameters, the mitotic index and presence of necrosis, observed by hematoxylin and eosin (H&E) staining, for the purpose of recognizing the four different subtypes (Travis et al., 2015). However, lung NE tumors represent a wide spectrum of phenotypically distinct entities, sometimes difficult to differentiate even for an expert pathologist. The distinction of the different lung tumors is of primary relevance in selecting appropriate therapy (Melosky, 2018; Tsoukalas et al., 2018). For these reasons, new biomarkers are needed in order to distinguish NE from non-NE lung tumors. miRNAs are a promising new class of cancer biomarkers which may potentially affect all aspects of clinical care from early detection, diagnosis, and prognosis (Du et al., 2010; Mallick et al., 2010; Detassis et al., 2017), to the discernment of site of origin in patients presenting metastaticity from an unknown primary tumor (Rosenwald et al., 2010; Gao et al., 2011). miRNAs are short non-coding single-stranded RNAs acting at the post-transcriptional level, dampening gene expression and, in turn, modulating cell behavior (Bartel, 2009). miRNAs analysis has been already proposed for the classification of lung tumors (Del Vescovo and Denti, 2015). Several works show that the relative quantification of miR205-5p in lung tumor biopsies and resected samples could be a diagnostic tool to correctly discriminate lung adenocarcinomas (AD) from squamous cell carcinomas (SQC) (Lebanony et al., 2009; Dacic et al., 2010; Sholl et al., 2010; Del Vescovo et al., 2011) and to sub-classify large cell carcinomas (LCC) according to their AD or SQC differentiation lineage (Barbareschi et al., 2011). However, little is known about the diagnostic value of the expression of miR205-5p and other miRNAs in identifying other lung tumor types such as the NE. The different subtypes of NE lung tumors show significant differences in their miRNAs expression profile (Mairinger et al., 2014), and it is known that miR375-3p alone can induce NE differentiation in cell lines and it is required to elicit ASH1-induced NE features via targeting YAP1 (Nishikawa et al., 2011). The basic helix-loop-helix protein, achaete-scute homologue 1 (ASH1), a master regulator of pulmonary NE cell development, is crucially involved in the pathogenesis of lung NE tumors. It is typically expressed by lung NE tumor (Demelash et al., 2012) and directly transactivates miR375-3p in cell lines and tumors (Nishikawa et al., 2011). In the present study we analyze miR375-3p expression in 95 surgically resected lung tumors, including 31 TC, 11 AT, 11 LCNEC, 4 SCLC, 22 AD, and 16 SQC and we demonstrate that, via an implemented statistical approach which has been recently developed and validated by our group (Ricci et al., 2015), miR375-3p is able to distinguish low-grade NE from non-NE lung tumors, but not LCNEC from SCLC tumors.

## Materials and Methods

### Histological Material

A series of 95 surgically resected and formalin-fixed and paraffin-embedded (FFPE) samples were collected between 1981 and 2011 by the Units of Surgical Pathology of the hospitals of Trento, Reggio Emilia, Forlanini-Rome and Modena (Italy). The samples had been stored at and were retrieved from the archives of Trentino Biobank,^1^ a structure based at the Santa Chiara Hospital, Trento, Italy. Trentino Biobank was established with resolution number 2007-S143-00261 of Trentino Autonomous Province (Provincia Autonoma di Trento) and resolution number 2008-890 of the Local Health Authority (“Azienda Provinciale per i Servizi Sanitari, Provincia Autonoma di Trento”). Trentino Biobank holds generic ethics approval and warrants to researchers access to the samples upon scientific review of the project by the Biobank Management Board. The samples included 31 TC, 11 AT, 11 LCNEC, 4 SCLC, 22 AD, and 16 SQC. All cases were fully anonymized, and the study has been approved by the Ethical Committee of the Santa Chiara Hospital, Trento. All slides of the tumors have been reviewed, and one representative paraffin block, with high tumor cell content, was selected for each case and used for RNA extraction. NE features of the tumors have been confirmed using an appropriate panel of immunohistochemical markers, including chromogranin A, synaptophysin and CD56.

### RNA Extraction

Four 10 μm sections were cut from the selected paraffin tissue blocks, placed in xylene and heated at 50°C for 13 min. The tube was centrifuged at 12,000 × *g* for 2 min, and the xylene was decanted. Residual xylene was extracted by the addition of 100% ethanol to the dewaxed tissue sections and centrifugation at 12,000 × *g* for 5 min was performed. The ethanol was removed and the process was repeated once. The samples were then air-dried for 30 min at room temperature. The Recoverall kit (Applied Biosystems) was used to extract total RNA from dried sections. This procedure involves DNase treatment, purification, and RNA elution. All samples were stored at −80°C until used for analysis. The concentration of each sample (ng/μl) along with the purity ratio (O.D: 260/280) was determined using a NanoDrop Spectrophotometer ND-3300 (Thermo Scientific).

### RT-qPCR

Quantification of miRNAs expression was carried out using TaqMan MicroRNA Assay kits according to the manufacturer’s protocol (Applied Biosystems). Prefabricated TaqMan MicroRNA Assays (containing miRNA-specific forward and reverse PCR primers and miRNA-specific Taqman MGB probe) were used for the investigation of miR21-5p (ID 000397), miR205-5p (ID 000509), miR375-3p (ID 000564), and RNU6B (ID 001093). RNU6B was used as an endogenous control to normalize miRNAs expression. Complementary DNA was generated using the TaqMan MicroRNA Reverse Transcription (RT) Kit (ABI P/N 4366596) according to the manufacturer’s instructions. Reverse transcriptase reactions contained 10 ng of total RNA as the template, 5 μL of gene-specific stem-loop RT primer, 1.5 μL of 10 × RT buffer, 0.15 μL of 100mM dNTPs, 1 μL of MultiScribe reverse transcriptase, and 4.16 μL of nuclease-free water. The 15-μL reactions were incubated on a Labcycler (SensoQuest GmbH) for 30 min at 16°C, 30 min at 42°C, 5 min at 85°C. Quantitative PCR was carried out using the CFX384 Touch^TM^ Real-Time PCR Detection System (BIORAD). The 20-μL PCR reactions contained 1.33 μL of RT product, 10 μL of FastStart TaqManProbe Master (ROCHE, P/N 04673417001), 7.67 μL of nuclease-free water, and 1 μL of TaqMan MicroRNA Assay (Applied Biosystem). Reactions were incubated at 95°C for 10 min, followed by 40 cycles of incubation at 95°C for 15 s and at 60°C for 1 min. The threshold cycle data (Ct) and baselines were determined using auto settings. All measures were done in technical triplicates and negative controls were included in each assay. Statistical analysis and technical outlier identification were performed as described elsewhere (Ricci et al., 2015). Briefly, technical outlier identification and statistical analysis were performed according to a pipeline developed within our research group: first, outliers are identified by checking their variability via chi-square test as well as their deviation by the respective population mean via Student’s *t*-test; second, on the basis of a training set of data, a Bayesian classifier is implemented which, by relying on the fact that triplicate expression averages are proven to be normally distributed, can be optimized and characterized also in terms of prediction uncertainty. For more details, the R package is available here: https://github.com/LeonardoRicci/MiRNA-QC-and-Diagnosis.

## Results

### Patients’ Cohort

At the time of this analysis, not for all the patients the clinical data of sex, age and cancer stage were available (23 out of 95 missing partial clinical data), due to the age of the stored samples and difference in the procedure of managing and saving clinical data among different hospitals. The patients cohort showed no statistical difference in the median age between TC, AT, AD, SQC, LCNEC and SCLC patients (All = 67.2 ± 5.9; TC = 54 ± 10; AT = 67.4 ± 13.9; AD = 69 ± 9.2; SQC = 70 ± 11.1; LCNEC = 64 ± 11.2; SCLC = 67 ± 9.7). The cohort was more represented by males than females (61M VS 17F). Clinical stage for each patient was considered following AJCC criteria (AJCC, 2020): IA1 = 7; IA2 = 11; IA3 = 5; IIA = 3; IIIA = 11; IB = 18; IIB = 11; IIIB = 5; IVB = 1 (Supplementary Table S1).

### miR205-5p Is Not Able to Classify Non-neuroendocrine From Neuroendocrine Lung Tumors

miR205-5p has been widely described as a good biomarker for discriminating AD from SQC (Lebanony et al., 2009). We have previously evaluated the discriminatory power of miR205-5p for AD and SQC lung tumors (Ricci et al., 2015) establishing the optimal score (ΔCt_205_) as follows:

$$\Delta \,{\text{Ct}}_{205}=\left({\text{Ct}}_{\mathrm{miR205}}-{\text{Ct}}_{\mathrm{U6}}\right)-0.8\times \left({\text{Ct}}_{\mathrm{miR21}}-{\text{Ct}}_{\mathrm{U6}}\right)$$

In the present work, we wondered whether ΔCt_205_ was able to discriminate also between non-NE and NE lung tumors. For this purpose, we evaluated the ΔCt_205_ in a cohort of 21 AD, 14 SQC, 6 AT, and 21 TC for a total of 35 non-NE and 27 NE samples. As depicted in Supplementary Figure S1, ΔCt_205_ is not able to sharply define non-NE from NE samples.

### Classifier for Low-Grade Neuroendocrine Lung Tumors

For the purpose of building a classifier to distinguish low-grade NE from non-NE lung tumors, we expanded the cohort for a total of 31 TC, 11 AT, 22 AD, and 16 SQC. We divided the cohort in a training and validation set and we measured U6 and miR375-3p expression by RT-qPCR TaqMan based technology. The training set included 27 non-NE (14 AD and 13 SQC) and 31 low-grade NE (8 AT and 23 TC). Each measurement was done in technical triplicates, and all the samples passed through a statistical reliability check (see section “Materials and Methods”) (Ricci et al., 2015). Using the average Ct we calculated, for each sample, ΔCt_375_ as:

$$\Delta \,{\text{Ct}}_{375}={\text{Ct}}_{\mathrm{U6}}-{\text{Ct}}_{375}$$

**FIGURE 1:**
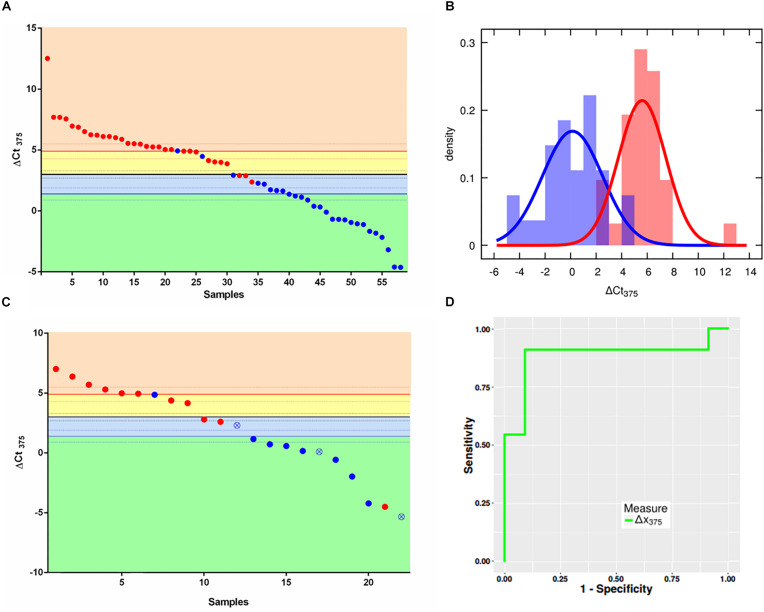
ΔCt_375_ analysis of the training set and validation set samples. **(A)** Scatter plot for ΔCt_375_ analysis (Ct_u6_–Ct_375_) of the training set samples: color-code divides the low-grade NE (red) and non-NE (blue). The quantity ΔCt_375_ is able to discriminate low-grade NE from non-NE with 92.6% of sensitivity and 90.4% of specificity. **(B)** Probability density functions of ΔCt_375_ relative expression for low-grade NE (red) and non-NE (blue). **(C)** Scatter plot for ΔCt_375_ analysis of the validation set samples: color-code divides the low-grade NE (red) and non-NE (blue). Empty-crossed circles represent technical outliers. **(D)** ROC curve for ΔCt_375_ which results in an AUC = 0.88.

Supplementary Figure S2 shows how U6 is stable in both groups analyzed bearing optimal characteristics as normalizer. The scatter plot in Figure 1A depicts in *x*-axis the samples ordered with a decreasing ΔCt_375_ (*y* axis), as well as the three thresholds χ10:90 (where 10:90 represents the odds of the sample of being non-NE or NE), χ, χ90:10. The two colors represent the two different classes assigned via immunohistochemical analysis and gene profiling by the pathologist: the non-NE lung tumors (blue) and low-grade NE lung tumors (red). The thresholds divide the plot into four boxes which reflect the different outcomes of the classifier based on the likelihoods:

• orange: AT-TC class with odds larger than 90:10 (ΔCt_375_ > 4,9)
• yellow: AT-TC class with odds between 50:50 and 90:10 (4,9 > ΔCt_375_ > 3)
• light blue: AD-SQC class with odds between 50:50 an 90:10 (3 > ΔCt_375_ > 1,4)
• green: AD-SQC class with odds larger than 90:10 (ΔCt_375_ < 1,4)

The accuracy is equal to 91.4%. However, if only high-reliable responses are considered, namely with odds at least 90:10 the accuracy is 72.4%. Considering statistical analysis reported in Materials and Methods, a maximum accuracy of 90.3% can be predicted for miR375-3p as Bayesian classifier. Figure 1B shows the probability density histograms regarding the two assigned classes: the non-NE lung tumors (blue) and low-grade NE lung tumors (red), with the Gaussian curves that fit the data representing the probability density functions. The two groups are well-separated with a Student’s *t*-test *p*-value = 5,5e10^–13^. The reliability of miR375-3p as classifier can be inferred by considering the confusion matrix (Table 1) which reports the numbers of each tumors class samples per threshold-defined box. The quantity ΔCt_375_ discriminates between the two groups with 92.6% of sensitivity and 90.4% of specificity.

**TABLE 1:** Confusion matrix of the training set. The quantity ΔCt_375_ discriminates low-grade NE from non-NE with 91.4% of accuracy, 92.6% of sensitivity and 90.4% of specificity.

	Classification			
	
Diagnosis	AD+SQC		AT+TC	
		
	>90:10	90:10 > p > 50:50	> 90:10	90:10 > p > 50:50
AD	9	4	1	0
SQC	10	2	0	1
AT	0	0	0	8
TC	0	3	5	15

### Test of the Improved Classifier on an Independent Data Set

Once we set the thresholds with the training set, we tested ΔCt_375_ reliability on the validation set of samples (22): 11 non-NE (8 AD, 3 SQC) and 11 low-grade NE (3 AT and 8 TC). We performed RT-qPCR measures of miR375-3p and U6 and then calculated the ΔCt_375_. We plotted the results using the classifier thresholds calculated previously with the training set (Figure 1C). With the exception of 4 misclassified cases, the classification provided by the classifier coincides with the immunohistochemical diagnosis (Supplementary Table S2). There are also three technical outliers, based on statistical analysis (Ricci et al., 2015), which, however, are correctly classified by ΔCt_375_. The resulting ROC (Figure 1D) displayed an AUC equal to 0.88. Moreover, the results of a one-way analysis of variance (ANOVA) on the data allow to state that ΔCt_375_ is not correlated with the clinical stage (Supplementary Figure S3), both for NE (*p* > 0.2) and non-NE (*p* > 0.05).

### Classifier Test for High-Grade Neuroendocrine Lung Tumors

**FIGURE 2:**
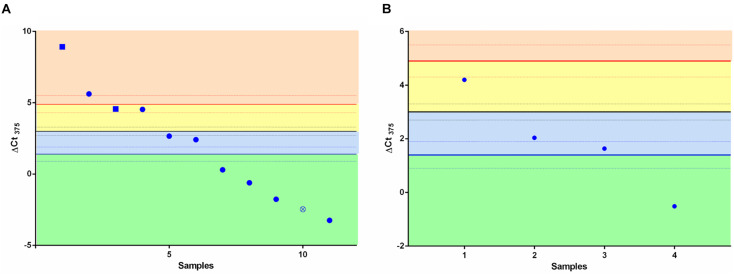
Scatter plots for the ΔCt_375_ analysis of LCNEC and SCLC samples. Neither LCNEC **(A)** nor SCLC **(B)** samples may be sharply discriminated by the ΔCt_375_ (Ct_U6_–Ct_375_) since they do not cluster in any of the ΔCt_375_ defined boxes (green and light blue: non-NE; yellow and orange: low-grade NE). Empty-crossed circles and full squares represent technical outliers (see Ricci et al., 2015).

We speculated whether the ΔCt_375_ classifier was able also to discriminate LCNEC (Figure 2A) and SCLC (Figure 2B) in either NE or non-NE lung tumors groups. We measured ΔCt_375_ in 11 LCNEC and 4 SCLC tissue samples and plotted the results according to the thresholds calculated previously. In both cases, the built classifier cannot divide either LCNEC nor SCLC in NE or non-NE type. However, considering the small cohort size, due to the rarity of such samples, the results are not conclusive.

## Discussion

Lung cancer is still a worldwide leading cause of death (Siegel et al., 2018). The treatments for this disease are different and vary depending on the type of lung cancer. In the era of personalized medicine, there is an increasing need for biomarkers and devices to classify the disease allowing proper treatment for each class of patients. miRNAs have been widely accepted as good biomarkers for several diseases, among which cancer. miRNAs are stable molecules well preserved in FFPE as well as in fresh snap-frozen specimens unlike larger RNA molecules as messenger RNAs. Being nucleic acids, they are easy to measure by RT-qPCR (Xi et al., 2007). It is well accepted that the expression of a set of miRNAs is a more reliable indicator of physiological or pathological changes, compared to one single miRNA. However, technological limitations, costs, and ease of use push toward the development of fast and immediate assays. In this sense, few miRNAs would be desirable for a diagnostic test compared to a whole set. For this reason, building an innovative collection of biomarkers requires a precise idea of its use and influence in clinical practice. miR375 has been shown to be involved in NE cellular development in several tumors (Abraham et al., 2016; Miller et al., 2016; Arvidsson et al., 2018). Post-transcriptional regulation of Notch signaling pathway and ASH1 make miR375 molecularly involved in NE differentiation (Nishikawa et al., 2011; Abraham et al., 2016). Interestingly, miR375 has also been demonstrated to be a good biomarker of diabetes, however, it is not clear whether the increase of the levels of miR375 is due to autoimmune mechanisms or pancreatic beta-cells destruction. A recent work, showed that the major part of the miR375 in circulation from NE cells in adrenal gland, the thyroid, the lungs and the gastrointestinal tract (Latreille et al., 2015; Eliasson, 2017). Our present study shows that using a mathematical approach based on a Kolmogorov-Smirnov statistic for the outlier classification and a Bayesian index it is possible to distinguish low-grade NE from non-NE lung tumors, based on the levels of miR375-3p (ΔCt_375_), with an 88% accuracy. This marker improves the differential diagnosis between non-NE and low-grade NE lung tumors which may be particularly challenging in small biopsies. Another level of complexity is given by the existence of different types of NE subsets. Reproducible and objective pathologic criteria with clinical and prognostic value must be established when comparing the various grades of pulmonary NE tumors (Travis et al., 1998). Additionally, there are no specific immunohistochemical or molecular markers that allow for separation of these tumors in clear groups. So far, some markers like Ki-67 are used to separate the high-grade SCLC and LCNEC and TTF-1 for LCNEC and basaloid squamous carcinoma, otherwise easily confused by morphology. However, Ki-67 is not very efficient in classifying AT vs TC tumors (Travis et al., 2015). Hence, in our work, we also investigated the possibility to discriminate high-grade NE lung malignancies by the measurement of ΔCt_375_. However, due to the biological differences of these tumors compared to the low-grade cases, the analysis was not relevantly proficient. The molecular complexity of these tumors (Rekhtman et al., 2016) dampens the capability of a clear classification. Thus, the pathologist pre-classification may not be able to cluster in clear groups the different tumor samples. The quantity ΔCt_375_ was tested on such pre-classification of high-grade NE lung tumors and its failure in subsetting the LCNEC and SCLC samples may also be due to the high molecular heterogeneity of these tumors. In this study we used FFPE samples being part of a standard procedure in clinical settings, however, liquid biopsies are becoming relevant for ease of use and patients’ compliance. Thus, in a future study of ΔCt_375_ ability in classifying NE from non-NE lung tumors, may be of critical value the use of plasma or serum. Moreover, a higher number of LCNEC and SCLC samples may increase the robustness of our findings in which miR375-3p is not able to distinguish among NE lung tumors. Concluding, here we report a miRNA marker which may fulfill the unmet need of the pathologists in clinical settings for the discrimination of NE from non-NE lung tumors also from small and challenging biopsies, making our findings of practical relevance.

## Data Availability Statement

The datasets generated for this study are available on request to the corresponding author.

## Ethics Statement

The studies involving human participants were reviewed and approved by Ethical committee of Santa Chiara Hospital. The patients/participants provided their written informed consent to participate in this study.

## Author Contributions

MD, VD, MG, MB, GR, PG, and AC designed the experiments. VD, MG, SM, SD, CC, and LR performed the experiments. MB, GR, PG, AC, and LC collected the samples. SD, VD, MG, and MD wrote the manuscript. SD and MD revised the manuscript.

## Conflict of Interest

The authors declare that the research was conducted in the absence of any commercial or financial relationships that could be construed as a potential conflict of interest.

## Footnotes

1 www.tissuebank.it
