# Supplementary material for: miR375-3p Distinguishes Low-Grade Neuroendocrine From Non-neuroendocrine Lung Tumors in FFPE Samples
Source: Front Mol Biosci. 2020 May 19;7:86. doi: 10.3389/fmolb.2020.00086 (PMC7263060; doi:10.3389/fmolb.2020.00086)
Supplement: Supplementary file 1 [file Table_1.docx]

**
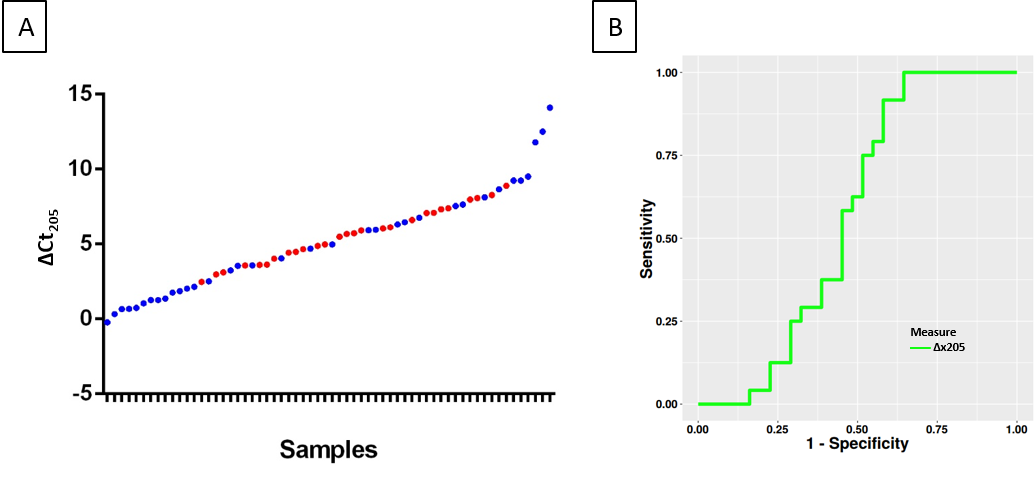
**

Figure S1. ΔCt_205_ for discriminating non-NE from NE lung tumor samples. (A) ΔCt_205_ ((Ct_miR205_-Ct_U6_) – 0.8 × (Ct_miR21_-Ct_U6_)) is not able to discriminate between non-NE samples (blue dots) and NE samples (red dots). (B) ROC curve of ΔCt_205_ with an AUC of 0.56

**
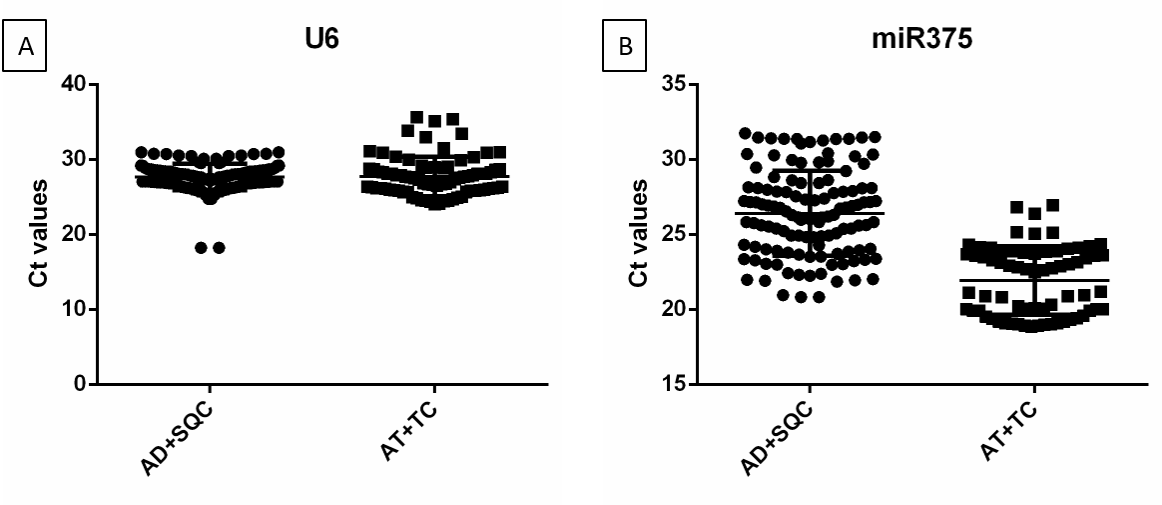
**

Figure S2. Ct values of U6 and miR375 for each analyzed sample. (A) U6 is stable among low-grade and non-NE samples indicating to be a good normalizer, while (B) miR375 shows variability between the two groups.





Figure S3. ΔCt_375_ divided per clinical stage. There is no significant difference correlation between ΔCt_375_ and clinical stage (ANOVA test: NE in red (p > 0.2) and non-NE in black (p > 0.4)).

**
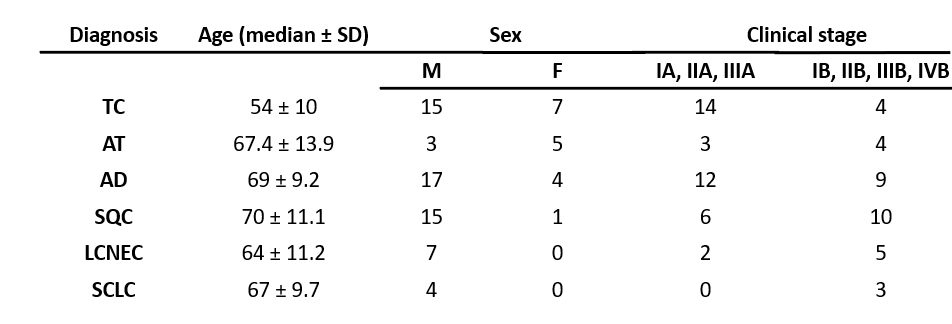
**

Table S1. Patients’ cohort divided for diagnosis, age at the time of diagnosis, sex and clinical stage following AJCC criteria.


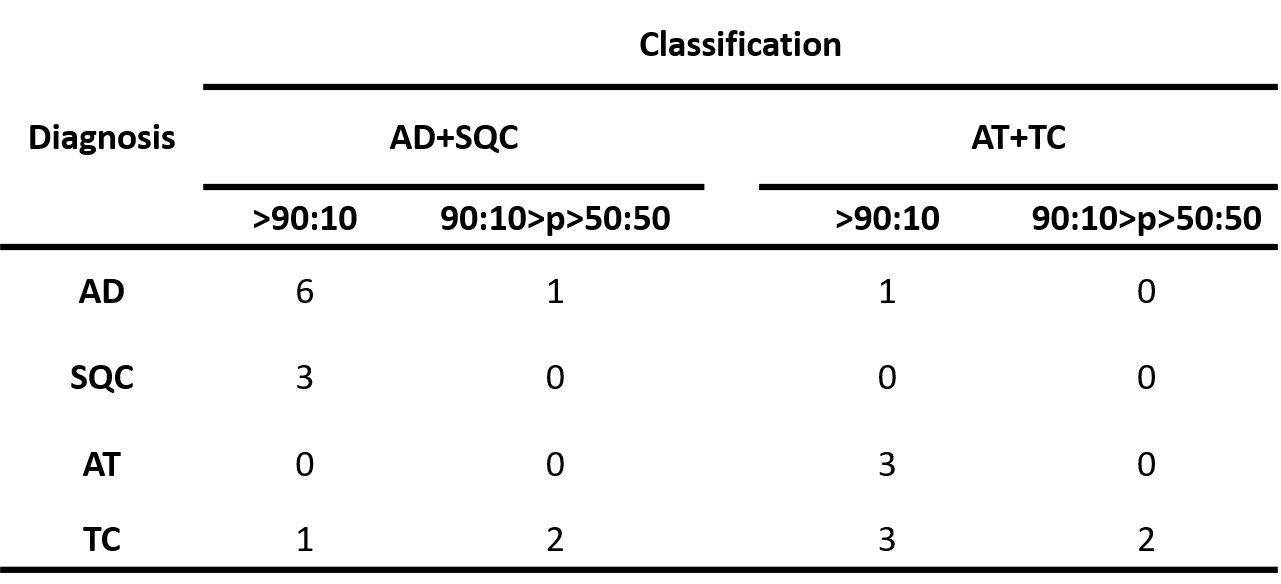


Table S2. Confusion matrix of the validation set. The quantity ΔCt_375_ discriminates low-grade NE from non-NE lung tumors with 91.4% of accuracy, 90.9% of sensitivity and 72.7% of specificity.
